# Deciphering the Genome Sequences of the Hydrophobic Cyanobacterium Scytonema tolypothrichoides VB-61278

**DOI:** 10.1128/genomeA.00228-15

**Published:** 2015-04-02

**Authors:** Abhishek Das, Arijit Panda, Deeksha Singh, Mathu Malar Chandrababunaidu, Gyan Prakash Mishra, Sushma Bhan, Siba Prasad Adhikary, Sucheta Tripathy

**Affiliations:** a Structural Biology and Bioinformatics Division, CSIR, Indian Institute of Chemical Biology, Kolkata, West Bengal, India; b Fakir Mohan University, Vyasa Vihar, Nuapadhi, Balasore, Odisha, India

## Abstract

Scytonema tolypothrichoides VB-61278, a terrestrial cyanobacterium, can be exploited to produce commercially important products. Here, we report for the first time a 10-Mb draft genome assembly of S. tolypothrichoides VB-61278, with 214 scaffolds and 7,148 putative protein-coding genes.

## GENOME ANNOUNCEMENT

Cyanobacteria are commercially important organisms that can be exploited in several sectors, such as pharmaceuticals and the fuel industry (1, 2). Moreover, these organisms are amenable to genetic engineering, so recently, a tremendous amount of importance has been given to this group. Scytonema tolypothrichoides VB-61278, a cyanobacterium, occurs in subaerial habitats, especially as biofilms on stone and mortar monuments and sculptures. It is hydrophobic in nature and grows well on solid substrates. Morphologically, S. tolypothrichoides VB-61278 is a greenish, filamentous, and heterocystous species. It reproduces by trichome axis fragmentation, and cell division happens in a clockwise direction relative to the trichome axis (http://www.cyanodb.cz). Some species of Scytonema are known to produce drugs used to treat viral diseases (3), HIV (4), bacterial diseases (5), and sometimes cancer (6).

S. tolypothrichoides VB-61278 was isolated from the exteriors of stone monuments in Santiniketan, West Bengal, India, and was grown on BG11 medium (7). Cultures were maintained at the Indian Institute of Chemical Biology under 16-h light/8-h dark conditions at room temperature (~28°C), with occasional shaking (once per day). The genomic DNA was extracted and purified using the UniFlex bacterial DNA isolation kit (Genei, USA). A total of 4 µg of genomic DNA was provided for sequencing. Two different types of libraries were constructed, a paired-end library with a 300-bp insert size and a mate-pair library with a 3-kb insert size. Sequencing was done using an Illumina HiSeq platform. For the paired-end library, the sequenced read length was 151 bases at 124× coverage (approximately 8.4 million reads). For the mate-pair library, the read length was 101 bases, and the coverage was 60×, generating approximately 6.5 million reads. The reads were cleaned using SGA (8) and TagDust (9), and the filtered high-quality reads were assembled using the AllPaths LG-49856 assembler (10). The final assembly was 10,008,488 bases in length, with 214 scaffolds. The largest and the smallest scaffolds were 1,436,913 and 5,004 bp, respectively, with an *N*_50_ value of 636,301 and a total G+C content of 47%. The assembly was annotated with the NCBI PGAAP (http://www.ncbi.nlm.nih.gov/genome/annotation_prok/). The total number of protein-coding genes is 7,148, the number of pseudogenes is 1,100, and there are 12 clustered regularly interspaced short palindromic repeat (CRISPR) arrays, 13 rRNAs, 80 tRNAs, 1 noncoding RNA (ncRNA), and 117 frameshifted genes predicted from this assembly.

The biosynthesis of the siderophore group of nonribosomal peptide synthetic pathways was predicted using KAAS-KEGG (11). The enzymes necessary for salicylate biosynthesis, isochorismate synthase, isochorismate pyruvate lyase, DhbF, and EntF are present in this assembly version. Eight genes from β-lactams and 7 genes from vancomycin resistance pathways were predicted in this genome. This organism also poses unique evolutionary challenges, and taxonomists opine that Tolypothrix and Scytonema should be merged (http://www.algaebase.org). The genome sequences of S. tolypothrichoides VB-61278 will undoubtedly be a very rich resource for biologists, genomicists, and evolutionary biologists.

### Nucleotide sequence accession number.

The whole-genome and annotation data of S. tolypothrichoides VB-61278 were submitted to GenBank under the accession no. JXCA00000000.
